# Promotion and prevention in child mental health

**DOI:** 10.4103/0019-5545.49447

**Published:** 2009

**Authors:** P. C. Shastri

**Affiliations:** Department of Psychiatry at B.Y.L. Nair Hospital and Topiwala National Medical College (Rtd.), Mumbai, India

## INTRODUCTION

The importance of psychological well-being in children and adolescent, for their healthy emotional, social, physical, cognitive and educational development, is well-recognized. There is now increasing evidence on the effectiveness of interventions to improve children's and adolescent's resilience, promote mental health and treat mental health problems and disorders.

Mental health problems will contribute significantly to the global burden of disease in the 21st century, and for adolescents, mental health hurdles are already as common as some physical health problems such as asthma.[1]

Clinical preoccupation of the available mental health professionals of the country and the delay of these professionals to spearhead work towards promotion, prevention, identification and early intervention in child mental health has been a major lacuna.

There are limited child and adolescent mental health services in India. Mostly such services are restricted to urban areas. Access to mental health services for children with a mental, emotional or behavioural disorder is substandard, not provided early enough, in sufficient supply and accessible only to a fraction of children and adolescents.

We currently have tertiary care centres which attend to mental illness in hospital setting. They are therapeutic in nature and aim to treat and rehabilitate back to society. However, large gap exists in the area of prevention, mental health promotion and early intervention programmes. Child Mental Health Policy and School Mental Health Programmes have provided excellent opportunity to enhance mental health programme for children and adolescents. Focus is rightly on preschool children and school based mental health programmes which will prevent and possibly promote positive mental health. It also ensures that it will reduce behaviour disorders in children and prevent adult psychopathology. Effectiveness of child mental health intervention programmes will surely help in addressing mental health disorders among adults.

Even WHO identifies the treatment gap in mental health care. World Health Organisation, asserts that many people suffering from psychiatric illnesses remain untreated, although effective treatment exists. WHO report examines the extent of this gap between the prevalence and treatment of psychiatric disorders globally. One in every 5 child has a mental health issue. If we invest in identifying the problems early and intervene at the right time, it will be more cost effective, as we will be preventing further breakdown and avoid an adult treatment and rehabilitation programme which is much more expensive. As it is rightly said that “Prevention is Better than Cure”. It is possible to prevent the majority of behaviour disorders in preschool and school environment itself.

The dire need is to stimulate long-term and sustained improvement in children's health, by setting standards for high quality integrated health and social care for children from before birth, right through to adulthood. And while doing this one of the main focus area has to be the mental health and psychological wellbeing of children and young people.

In order to achieve desired outcomes one should embrace all those services that contribute to the mental health care of children and adolescents, whether provided by health, education, social services or other agencies. It is also crucial to partner with services whose primary function is not mental health care, such as GPs and schools. They can always contribute by offering general advice and treatment for less severe problems, contribute towards mental health promotion, identify problems early in their development, and refer to more specialist services. This is to explicitly acknowledge that supporting children and adolescents with mental health problems is not the responsibility of specialist services alone.[2]

The plan should include primary mental health workers, psychologists and counselors working in GP practices, pediatric clinics, schools and youth services. They can offer consultation to families and other practitioners, outreach to identify severe or complex needs which require more specialist interventions, assessment, treatment and training where needed.

We need a multi-disciplinary team or service working in a community mental health clinic or child psychiatry outpatient service, providing a specialised service for children and adolescents with more severe, complex and persistent disorders. This team can include child and adolescent psychiatrists, social workers, clinical psychologists, community psychiatric nurses, child psychotherapists, occupational therapists, art, music and drama therapists.

Mental health service delivery demands effective partnerships between agencies, joint protocols to be agreed at senior officer level between the local, state and national bodies, social service organizations and educational institutions. There should be a single window operation in order to develop an effective model for child and adolescent mental health.

Also services for children and adolescents should be provided irrespective of their gender, race, religion, ability, culture or sexuality.

Traditionally in India, the responsibility of care and protection of children has been with families and communities. A strong knit patriarchal family that is meant to look after its children well has seldom had the realization that children are individuals with their own rights. Child and Adolescent Mental Health is the fundamental right of the children, and the approach to ensure the fulfilment of these rights so far has always been more need based rather than rights based.

A typical Indian child starts his/her life in the womb with intrauterine growth retardation (30 percent) due to factors like malnutrition and anaemia. The child especially a female child continues to have deprivation and discrimination all through his/her life. Indian child undergoes multidimensional exploitation inflicted both at home and work-place; economically, sexually, personally and educationally. This results in poor identity and self-worth of the child. Even the legislative and social changes through mass movement on community awareness in the direction of compulsory schooling, have failed to ensure mental health to an Indian child.

The Nation's children are a supremely important asset. Their nurture and solitude are our responsibility. Children's programmes, should find a prominent part in our national plans for the development of human resources in each sector, so that our children grow up to become responsible citizens. Equal opportunities for development to all children during the period of growth should be our aim, for this would serve our larger purpose of reducing inequality and ensuring social justice.

## CURRENT INDIAN SCENARIO

According to the UNESCO report 2008. (U.N.D.P. REPORT 2008):[3]

India stands at

- 102^nd^ position in the “Education for all developmental index" out of 129 countries”.
- 132^nd^ place in the list of 172 nations on human development index (H.D.I)

Ten percent of 5-15 year olds have a diagnosable mental health disorder. This suggests that around 50 million children under eighteen would benefit from specialist services. There are up to 20 million adolescents with a severe mental health disorder. Around 90% of children with a mental health disorder are not currently receiving any specialist service.

All the elements that play key role in a child development and mental health are in unattended state in India. This includes basic amenities, poor infrastructure, and human resource development along with essential health and hygiene measures.

Mental health promotion strategies need approaches that are also associated with the prevention of child and adolescent problems within communities with low Socio-economic Status (SES). Low SES can be measured in different ways including low levels of education and/or income or definitions that combine several variables. This is mainly because the factors associated with low SES are also associated with the development of violence and crime, substance abuse and child health problems. Interventions that address underlying determinants of low SES show strong efficacy in decreasing adolescent crime and violence and effectiveness in improving child health outcomes. Infact there are programs designed to improve educational pathways that show some efficacy in reducing aspects of adolescent substance use. Such interventions could also be supported in mental health promotion policy as they may assist in preventing related problems that undermine mental health.

Following areas need attention for promotion, prevention, identification and intervention:

- Promoting Health and Well-being, Identifying Needs and Intervening Early
- Supporting Parenting
- Child, Adolescent and Family-centered Services
- Growing Up into Adulthood
- Safeguarding and Promoting the Welfare of Children and Adolescents
- Children and Adolescents who are Ill
- Children and Adolescents in Hospital
- Disabled Children and Adolescents and those with Complex Health Needs
- The Mental Health and Psychological Well-being of Children and Adolescents
- Medicines for Children and Adolescents
- Antenatal, Peri-natal and Postnatal Services

‘Primary care’ is of crucial importance and includes all first line services that have contact with children and their families.

## NATION'S ATTEMPT TO ADDRESS THE ISSUES

The major national policies and legislations formulated in the country to ensure child rights and improvement in their status include:

- National Policy for Children, 1974
- National Policy on Education, 1986
- National Policy on Child Labour, 1987
- National Nutrition Policy, 1993
- National Health Policy, 2002
- National Charter for Children, 2004
- National Plan of Action for Children, 2005

However, there is a wide gap between identifying needs, planning, developing policies and effective implementation to bring a difference. But there is hope when concerned authorities continue to take the matter seriously and address the preliminary rights that aid in child mental health issues.

“National Human Rights Commission will guard right to education and health. Focus will be on life, survival, health and basic education. Elementary education and primary health services in rural areas will get top priority.”

- S. Rajendra Babu

Former Chief Justice of India, Chairperson of NHRC

September 2007

## ACTION PLAN

### Prevention

Effective prevention programs have been identified which may help to reduce the risk of children developing a mental problem or disorder. Some prevention programs are even more effective than later treatments, particularly in the area of conduct disorders. Significant advancements can be made when both the early years of life and the early stages of disorders are targeted.[4] Mental health prevention and early intervention are relatively new fields in mental health. Progression of these initiatives involves supporting health and related staff and the community in the acquisition of the knowledge and skills needed to meet the challenges of new service directions and programs, including the provision of resources to assist implementation.

Child's mental health may be affected by events such as death of a family member, marital discord or separation, environmental disasters and economic disadvantage. Children and adolescents may require interventions to ameliorate the effects of abuse or neglect, parental substance abuse or mental health problems or domestic violence.[5–7]

Lack of appropriate stimulation in the early years may result in language delay and together with inappropriate child-rearing practices, especially if characterised by neglect or inconsistency, may lead to emotional or behavioural disorders. Appropriate parenting styles are fundamental to caring for children's mental health. Early attachment and bonding between parents and their babies is important and needs to be supported.

Many children, adolescents and their families who could benefit from mental health services for assessment and treatment are not accessing services. There are a variety of reasons for this: a lack of trust in statutory services; a wish to solve problems themselves; a lack of recognition and agreement that a problem exists; a fear of being teased and stigmatised; a fear of confidentiality being broken and a belief that nothing can be done. These can all affect the take-up of help.

Children and adolescents rarely present with single disorders but rather with a range of problems. A large proportion of the available evidence does not reflect the co-morbidity issues which present in day-to-day clinical practice. In addition, services have to rely frequently on either extrapolating research findings from abroad or from adult literature.

When it comes to prevention, one must give necessary attention to genetic causes, environmental factors and the interaction between the two that can cause several childhood disorders, as some of these are preventable.[6] Advancement of genetic techniques aid in prenatal diagnosis and have importance in counseling including premarital counseling. For e.g. early identification of PKU would prevent mental retardation. Cretinism, Iodine deficiency and malnutrition are also easily correctable and preventable conditions.

### Promotion

All children, adolescents and their parents or carers require access to information and supportive environments to ensure that the child or adolescent's mental health is promoted. Specific activities such as tackling bullying, provision of education to increase awareness of mental health issues and to improve the recognition of children's emerging needs, and provision of support for those children with particular needs, have a vital role to play in improving the chances for children and adolescents. Everyone in a community has a role to play in ensuring that the environment in which children are growing up promotes their mental health.

For children with learning difficulties and their parents or carers, the provision of special education, training and promoting need for early intervention may make a significant difference in overall development.

One should invest time and resources in refocusing of services that will be necessary to meet their needs. This will include ensuring that there is a strong focus on vocational and social issues in order to ease adolescent's transition into adulthood and reduce the likelihood of social exclusion, so often a secondary consequence of mental illness.

Assessment of local needs may identify other groups of children and adolescents for whom service development is required e.g. looked after children, where there has been recent significant improvement in provision, children with conduct disorder or severe behavioural problems, children and adolescents who are homeless, adolescents in young offenders institutions and asylum seeking children, where expertise is not readily available.

Given that some forty per cent of children with learning disabilities have a diagnosable mental disorder and this rate is even higher in those with severe learning disabilities, the low level of resources available to the children and their families represents serious inequity and a significant challenge for the development of appropriate services.

There is normally a wide variation in the age when adolescents achieve maturity and independence, especially for those with learning disability and other impairments. A degree of flexibility is clearly required to ensure that adolescents receive treatment in an environment that promotes their engagement and responds to their developmental needs.

A nurturing social environment in childhood, good early education and academic success in school are related to protecting the mental health of young growing generation. The influence of peers is also critical.

For good mental health services, the following five sectors are important:[8,9]

- Early years
- School years
- Community based activity
- Additional and support needs
- Children in need of special care

Child mental health services at district level should incorporate the following programs:[10,11]

- Liaison
- Consultation
- Training
- Supervision
- Intervention
- Planning and development
- Research and development

Training should aim to consolidate existing knowledge through experiential learning, enabling staff to promote good mental health and recognise and manage children and adolescent's mental health problems at an early stage. Training should be appropriate to the developmental level and cultural context of the children and adolescent's population.

Primarily educative, supervision should aim to improve the ability of professionals to promote and support children and adolescent's mental health more effectively by improving their skills, knowledge base and facilitating reflection on attitudes towards mental health, thus enabling more effective practice. Supervision can take the form of individual or group support and can also act as a means of consolidating multi-agency training.

Children in most sections of Indian society are traditionally and conventionally not consulted about matters and decisions affecting their lives. In the family and household, the neighbourhood and wider community, in school or in work place, and across the settings of social and cultural life, children's views are mostly not given much importance. If they do speak out, they are not normally heard. The imposition of restrictive norms is especially true for a girl child. This limits children's access to information and freedom to choose, and often to the possibility of seeking help outside their immediate circle.

Child mental health is a shared responsibility, and for any intervention to be effective there should be a synergy between efforts being made by different stakeholders to address the issues. There is a need to create a mechanism that will make such a synergy possible. These may include child mental health prevention and promotion mechanisms at village, block, district and state levels which involve parents, elected representatives of urban and rural local bodies, teachers, *anganwadi* workers, medical practitioners, police and social workers and responsible members of public among others.

The media should be productively used to spread awareness on child mental health. Debates and discussions with participation of children can be a regular feature on electronic media in order to enhance people's knowledge and sensitivity on child mental health issues.

We also need appropriate and updated prevalence and incidence database from all the possible agencies for planning and implementation.

Children's voices need to be heard by everyone. All for addressing issues of child mental health should have adequate children's representation with the opportunity for them to express their views. For example, school curricula should be developed with the active participation of children; children should be involved in development of the district child protection plan, children should be involved in management of schools and institutions, etc. It is mandatory that peer education, peer training and peer participation should be part of each and every school mental health program.[12]

### Early identification

Mental health problems in children are associated with educational failure, family disruption, disability, offending and antisocial behaviour, placing demands on social services, schools and the youth justice system. Untreated mental health problems create distress not only in the children and adolescents, but also for their families and carers, continuing into adult life and affecting the next generation.

Some children in special circumstances have greater needs regarding their mental health. Looked after children are five times more likely than their peers to have a mental health disorder. Children and adolescents with significant learning disabilities are three to four times more likely to have a mental disorder and at least forty per cent of young offenders have been found to have a diagnosable mental health disorder.

Almost 20 per cent of all children and adolescents are affected by mental health problems and at least half of these show impaired schooling and social development.[13]

Among children and adolescents, problems such as child abuse and neglect, conduct disorders, alcohol and drug abuse, depression, attention deficit disorders, and suicide are all becoming more common.[14–16] Furthermore, mental disorders (notably depression) are appearing at a younger age and they also seem to be increasing in severity.[17,18] Children and adolescents with mental health problems are:

- Twice as likely to report feeling ‘very stressed’
- Three times more likely to have poor or fair physical health
- Three times more likely to perform below grade level at school
- Three times more likely to use alcohol and other drugs
- Six times more likely to think about killing themselves.[19,20]

## WHAT IS NEEDED IS TO SEE THAT

- All children and adolescent get best and improved mental health.
- Multi-agency services, working in partnership, promote the mental health of all children and adolescent, provide early intervention and also meet the needs of children and adolescent with established or complex problems.
- All children, adolescent and their families have access to mental health care based upon the best available evidence and provided by staff with an appropriate range of skills and competencies.

All children and adolescents, from birth to their eighteenth birthday, who have mental health problems and disorders, need to have access to timely, integrated, high quality, multi-disciplinary mental health services to ensure effective assessment, treatment and support, for them and their families.

## IN ORDER TO ACHIEVE WE MUST ENSURE THE FOLLOWING AT DISTRICT LEVEL

- All staff working directly with children has sufficient knowledge, training and support to promote the psychological well-being of children and their families and to identify early indicators of difficulty.
- System of referral, support and early intervention are well worked out.
- Professionals provide a balance of direct and indirect services and are flexible about where children and families are seen.
- Children and adolescents are able to receive urgent mental health care when required.
- Children and adolescents with both a learning disability and a mental health disorder have access to appropriate child and adolescent mental health service.
- Arrangements are in place to ensure that specialist multidisciplinary teams are of sufficient size and have an appropriate skill-mix, training and support to function effectively.

### Intervention

Similar numbers of children with less serious mental health problems will need some help. In most cases, this will be provided by services in primary health care, social care, education (including early years) and the voluntary sector.

In many cases, the intervention that makes a difference will come from another service. For example, a child presenting with behavioural problems may make better progress if his/her literacy problems are also addressed, in which case an input is required from education. The lack of provision in one service may impact on the ability of other services to be effective. Partnership working is an essential requirement of high quality service provision.

Concepts of mental health and illness and the understanding of the origins of children's emotional and behavioural difficulties vary across cultures. Services need to be sensitive to these differences and ensure that staff is equipped with the knowledge to work effectively with the different groups represented within the community they serve.

The experiences of those families who are refugees or are seeking asylum, particularly those from conflict torn zones, have often been highly traumatic. The provision of effective mental health care can be extremely challenging, especially if there are language barriers. For localities with a significant population of such families, specific arrangements may need to be made to provide appropriate mental health care for the children and adolescents within these families.[21,22]

While planning such services, proximity of the location is also important factor for consideration. This is also keeping legislative mandates in mind for provision of services and can be within 2 to 5 km distance. This is mainly advised so that they are able to access services easily. Time and day for availability of services should also be convenient to both child and family in order to make it effective and avoid drop-outs.[23]

The setting in which the first contact is made may make a difference e.g. in school which may be seen as less stigmatising for some or, where confidentiality is of particular concern, away from school for a young person who fears being teased.[24,25]

The development of services for children with learning disabilities will require a workforce with the competencies and knowledge of working with children and adolescents with complex, severe and multiple disabilities (especially those with moderate or severe learning disabilities) and their families. Co-ordination of learning disability with other services should be achieved through partnerships which preserve and enhance the quality and effectiveness of specialist provision already in place.

Partnership working across agencies working with children and adolescents with mental health problems can be a challenging task. The lack of understanding of the respective roles, duties, responsibilities and organisation of the different agencies and professionals and of their different language, may lead to poor communication, misunderstandings and frustration. Effective partnership working can improve children and adolescent's experience of services and lead to improved outcomes.[26]

A critical mass of staffing is required for services to be safe, timely and effective and able to respond to a wide range of demands which include the provision of: specialist and multi-disciplinary assessment and treatment services; teaching, specialist consultation and liaison services; research and audit; and support, training, consultation and face-to-face work within primary care settings.[27] The precise level of staffing will vary according to indices of deprivation, whether the service is in a rural or urban setting, the number of local partnerships required and teaching responsibilities. Where services have good core resources, they are also able to offer a range of specialist and community-out-reach services; this arises from the availability of a critical mass of staffing. Many services have not been able to recruit all members of a multidisciplinary team, which limits their capacity to provide a comprehensive service.[27,28]

Services ensure that children and adolescents receive treatment interventions which are guided by the best available evidence and which take account of their individual needs and circumstances.

Currently such services are majorly provided dedicatedly by NGOs and private institutions and professionals. There was a pervasive concern that while multiple public and private entities had important roles to play in meeting the mental health needs of children and families, there was an absence of overall comprehensive planning, accountability was as fragmented as the rest of the system, and as a consequence there was a sense that nobody was responsible at the national and government level.

An estimated 90% one in five of all adolescents with mental disorders is not receiving any treatment. This is a result of several factors. First of all services to address these don't exist. And also most often, children's and adolescents' mental health problems are not recognized or diagnosed properly, and available effective treatment is not employed.

Children and adolescents with mental health problems are most often handled by the school or juvenile justice systems, which are generally ill-equipped to recognize and address mental disorders.[29]

A number of treatment models have been found to be effective in addressing mental health problems of children and adolescents. These prevention and intervention strategies, however, are often underutilized. While they can require significant time and investment, many prevention and intervention models are economically efficient when compared to the cost mental health problems extract from youth, their families and communities.

Successful programs involve long-term intense interventions and address an array of factors such as family conflict, depression, social isolation, school failure, substance abuse, delinquency, and violence.

Intervention can also be provided by direct work with children and families, where the level of need appropriately matches the type of intervention normally provided in a primary care environment. Direct interventions should be brief and tailored to the child/ adolescent's and family's identified needs.[29]

Mental health promotion (MHP) and mental disorder prevention (MDP) among children and adolescents need to shift its focus. The change needed is to move from inherited patterns of institutionalization and medicalization to modern public health approaches based on involvement of children, youth, parents and communities.

Another big challenge in the field of child mental health is to plan and organize effective parenting programs for mentally ill parents. These programmes would need special skills and training to make it effective for both parents as well as children.

## PREVENTION, PROMOTION, EARLY IDENTIFICATION AND INTERVENTION: A SPECIALITY

Supporters of the population health approach to mental health have consistently advocated primary prevention of children's problems. However, awareness, education and training is required for recognising that childhood and youth constitute defined developmental phases, and that problems in this period are often interactive, contributing to the escalation of vulnerability to mental health problems or disorders. The aim of prevention and early intervention is to be able to alter this trajectory.[30]

Thus, it is essential that a comprehensive prevention agenda is built on an alliance of health, education and social agencies in our communities. For many health, education and community workers this requires a new way of thinking about mental health. The focus must shift from individual clinical casework to a broader population mental health understanding including:

- Epidemiology
- Multifactorial aetiology
- Risk and protective factors
- Socio-environmental determinants of health and mental health such as poverty and unemployment
- Socio-cultural processes.[31]

In particular, professionals employed in mental health services must be aware that in prevention, the proximal social environments that are most pertinent to population health problems are:

- Family
- School
- Workplace
- Media
- Social organisations
- Professional organisations
- Community organisations
- Peer and other social groups.[31]

## CONCLUSION

The country has to take care of an enormous number of children. While articulating its vision of progress, development and equity, India has expressed its recognition of the fact that when our children are educated, healthy, happy and have access to opportunities, they are the country's greatest human resource.

This will require commitment to the integrity of programs, their adaptation for and engagement with local communities, and the incorporation of evaluations of program effectiveness. More attention is now being given to the need for programs to provide quality norms for good practice that are determined by theory, evidence based outcomes, cost effectiveness and feasibility of widespread implementation. We must assist the implementation of innovative and effective mental health initiatives in this relatively new field in mental health services for children and adolescents across India.

India presents a unique case in terms of the sheer size of its population and 46 percent of them are children; characterized by heterogeneity in respect of physical, economical, social and cultural conditions. Its population of 1.12 billion constitutes 16 percent of the world population, with 74 percent of them living in rural areas. India is a secular state with various languages, cultures and religions. This kind of complex and multifaceted country makes formulation of National policies, programming and planning quite a challenging task. Each and every one of the 600 districts of India is unique in many ways. Each district will need its planning at local level. For such a diversified country it is difficult to envisage a national program that fits all and even of all are considered in reality it may fit none.

Independent India has taken large strides in addressing issues like child education, health and development. But, it has failed to implement program which is progressive, promotional, performance based, preventive and protective to the child mental health. Examining the government policies and national program for promoting child mental health it becomes evident that there is a wide gap between the children's needs and existing resources. There is neither an independent nor integrated child mental health policy in India. The multiple needs of a child are currently covered by different policies and subsequently different ministries. It is crucial to develop a comprehensive policy to cover all aspects of children's mental health, fewer than one umbrella.

One of such endeavour is the 11^th^ Five year plan, which is child centred plan. This plan includes a) Child mental health policy, b) School mental health policy and c) Mental health policy for disabled. We need to take maximum advantage to make use of these three policies where program and funds are structured and in favour of child mental health.

The incidence of children needing mental health services is high. Even after sixty one years of independence, resources to meet the mental health needs of children, manpower, as well as preventive, diagnostic and treatment services are extremely limited. Who is responsible for this gap in demand of such crucial child mental health services and meeting the need? Is it inadequate government policy and/or unaroused citizenry and/or insufficient resources and/or the lackadaisical attitude of people towards the needs of children? We must urgently introspect this in order to achieve future positive outcomes.

One thing is certain. Single window operation for child mental health, education and welfare will surely go a long way in successful implementation of various child legislations providing right control, quick results and ensuring justice for successful mental health programmes.
